# Embracing the heterogeneity of natural viruses in mouse studies

**DOI:** 10.1099/jgv.0.001758

**Published:** 2022-06-23

**Authors:** Jessica K. Fiege, Ryan A. Langlois

**Affiliations:** ^1^​ Department of Microbiology and Immunology and the Center for Immunology, University of Minnesota, Minneapolis, USA

**Keywords:** dirty mice, virome, microbiome

## Abstract

Animal models are a critical tool in modern biology. To increase reproducibility and to reduce confounding variables modern animal models exclude many microbes, including key natural commensals and pathogens. Here we discuss recent strategies to incorporate a natural microbiota to laboratory mouse models and the impacts the microbiota has on immune responses, with a focus on viruses.

## Introduction

Free-living animals are exposed to pathogenic and commensal micro-organisms throughout their lives. Animal models have been key to uncovering the biology of diseases and mice in particular have become an essential part of modern biology research. However, the majority of studies using the mouse animal model occur in specific pathogen-free (SPF) animals. These animals live in barrier housing and are protected from many pathogenic and commensal bacteria, fungi, protists, helminths and viruses that can infect mice, cause disease, complicate studies and decimate vulnerable colonies [1, 2]. An unintended side effect of SPF housing is an underdeveloped immune system compared to free-living animals. Many pre-clinical studies, which are beneficial in SPF animals, fail to be recapitulated in humans, sometimes with devastating results [1, 3, 4]. Recent studies have aimed to better understand the impact of the microbiome on the maturation of the immune system. However, these studies often focus on bacteria and helminths and the impact of viruses are less well characterized. Studies incorporating diverse microbial experiences into standard mouse models are needed. It is important to understand how trans-kingdom microbial species interactions contribute to the development, maintenance, and function of the immune system.

The microbiome can have wide-ranging impacts on their hosts from nutrient uptake and metabolism to immune development and protection from pathogenic infections. Much of the focus of these studies has been on the impact of prokaryotes. Here we will focus on viruses and their impact on mouse models. Viruses are obligate intracellular parasites that can be found in all domains of life. Despite the presence of viruses across all living things, eukaryotic viruses are often only viewed through the lens of pathogenic responses. However, an emerging body of work has demonstrated the beneficial roles viruses play in protecting the host from infection and in the development of the immune system. These studies are reshaping our view of how viruses interact with their hosts. Viruses can be commensals or pathogens and both of these physiologic states can impact the immune system at steady state and the responses to new infections. Introducing natural mouse viruses into traditional laboratory mouse experiments can help to uncover new roles for viruses in immune development and homeostasis, increase translatability of research findings, and can be used to study the transmission and evolution of viruses.

## Impacts of the virome on mice

Viruses are intracellular parasites impacting all living things from prokaryotes to humans. Immune systems have evolved a litany of diverse mechanisms to recognize and control invading viruses. Co-existence with viruses is a feature across all life, however eukaryotic viruses have largely been eliminated from standard laboratory animal models. Shortly after birth humans begin experiencing virus infections impacting the developing immune system [5–7]. Humans are also colonized with hundreds of species of prokaryotes, which carry phages with them. Phages are the most abundant and diverse virus species on earth and within living animals. While phages can have significant impact on immune responses in mice and humans, here we will only focus on eukaryotic viruses. A key facet of commensal organisms is that they can provide tangible benefits without causing disease in the host. There has been significant effort made into studying the impact of prokaryotic organisms on the development and maintenance of the immune system of mammals. The role of viruses within the microbiome has only recently begun to be appreciated. It is clear that eukaryotic viruses can infect their hosts without causing overt disease [2, 8–10]. The impact of these infections has been a burgeoning area of research. Studies in mice demonstrate persistent infection with murine norovirus without overt disease [11]. Similarly, astroviruses are present in many mouse colonies and in wild mice and go undetected not just because it is not screened for but also because it does not drive pathology [2, 8, 9, 12].

There is a growing body of literature suggesting that eukaryotic viruses can provide many of the same benefits as the prokaryotic microbiome to the host. Experiments to reduce virus replication in mice through antiviral drugs impacted broad facets of the immune system demonstrating the wide-ranging effects of viruses on basal immune responses [13–15]. Gnotobiotic mice that lack commensal micro-organisms have wide-ranging defects in immune system development. Introduction of murine norovirus can correct many of these immune abnormalities, acting as a surrogate for the role of commensal bacteria [11]. Cadwell and colleagues took this concept further by rigorously examining the impact of individual enteric viruses in wild-type and germ-free mice, demonstrating that individual viruses can provide some of the same basal signals for the immune system as the bacterial microbiome [16]. Single viruses can drive lymphocyte differentiation although individual viruses also drove many unique responses [16]. In some instances, the virome can protect from disease and promote tissue repair. Homeostatic levels of enteric viruses protect SPF mice against dextran sulphate sodium (DSS)-induced colitis [15]. Decreasing DNA and RNA viruses with an antiviral cocktail containing ribavirin, lamivudine and acyclovir followed by DSS-induced colitis resulted in enhanced colitis pathology [15]. Conversely, infecting mice with inactivated rotavirus prior to DSS-induced colitis protected animals from colitis, which is dependent on TLR3/7 signalling and the production of IFN-I [15]. The virome could protect through other mechanisms as well. Some virus infections have been demonstrated to promote tissue repair and wound healing [17]. For example, infection with human cytomegalovirus (hCMV) promotes the induction of a mesenchymal-to-epithelial transition phenotype [18], which is associated with healing of damaged epithelial structures and repopulation with healthy epithelial cells [19]. Additionally, CMV-infected cells secrete cytokines, chemokines and growth factors, which could also impact wound healing [17, 20]. The pathways that the host uses to respond to viruses can also impact host physiology. Antiviral signalling through RIG-I and MAVS promotes gut integrity, protecting against immune-mediated injury during graft-versus-host disease [21]. While the causative agent is unknown, this is ostensibly occurring because of the presence of eukaryotic viruses or phages. Together these studies demonstrate that viruses can provide critical cues to the immune system impacting homeostasis, disease pathogenesis, and responses to subsequent infections.

Viruses can also potentiate diseases not directly related to acute viral pathology. Reoviruses are common mammalian enteric viruses that can infect without causing intestinal damage. In the presence of dietary antigen, infection with reovirus induced a loss of oral tolerance in mice. Additionally in humans, coeliac disease patients exhibited elevated antibody levels against reovirus [22]. These data suggest that reoviruses can drive the induction of immune-mediated disease. Noroviruses serve as another example. Noroviruses are common in humans and other mammalian species and are the leading cause of gastroenteritis in both children and adults [23]. While noroviruses are generally acute, some strains can persist for months after initial infection. Persistent norovirus infection followed by DSS treatment in mice harbouring a mutation in *Atg16L1*, a Crohn’s disease susceptibility gene, resulted in inflammation and hallmarks of Crohn’s disease [24]. Interestingly, treatment with antibiotics or blocking TNF or IFNγ significantly reduced DSS-induced disease in *Atg16L1* mutant mice [24]. These data suggest that persistent norovirus infections can drive Crohn’s disease in susceptible hosts. Additionally, infection with DNA viruses, Epstein–Barr virus, human papilloma viruses, hepatitis B virus, human herpes virus-8 or Merkel cell polyomavirus, or RNA viruses, human T lymphotropic virus type I and hepatitis C viruses, among many others can contribute to cancer development in humans [25, 26]. Together these data demonstrate the indirect effect common viruses can have on disease.

Viruses can directly and indirectly impact the makeup of the virome and immune responses. For example, anticorrelations between astroviruses and noroviruses and astroviruses and coronaviruses have recently been uncovered [9, 12]. Interestingly, the makeup of the eukaryotic virome in infants can impact the immune response to, and replication of, live attenuated rotavirus vaccine [7]. There are several potential mechanisms for these virus-dependent inhibitions including induction of interferon and potentially cross-reactive antibodies for closely related viruses. Together these data suggest that acute and chronic viruses present in humans and mice can impact subsequent virus infections and these potential viral interferences should be considered when designing virus challenge experiments. Absence of a natural mouse virome can have major impacts on immune and viral pathogenesis studies.

## Models to introduce microbial experience

The mouse has become a ubiquitous feature of modern biology studies because of low costs, ability to genetically manipulate animals and the wealth of experimental tools. Mice housed in SPF conditions can reduce confounding variables from heterogenous pathogens, which improves reproducibility across laboratories. However, this practice also has several drawbacks, notably, loss of microbial immune experience and an altered, non-natural, microflora. Researchers have devised several distinct strategies to incorporate a more natural microbial life history while still leveraging the advantages of traditional laboratory mice. The benefits and drawbacks of these experimental designs with a focus on the impact of viruses will be discussed below.

## Wild mice

The study of wild mice has yielded discovery of multiple new viruses and a greater understanding of the variability in a wild immune system. Researchers have been testing wild mice for the presence of viruses by serology across the world for decades [27–31]. One difficulty in studying wild mice is controlling for timing and source of pathogen exposure. Wild mice captured in California, Virginia and New York City led to the identification of many novel viruses including murine astrovirus and Kobuvirus [2, 10, 32]. Evaluation of wild mice has also revealed complex trans-kingdom interactions. For example, Lipkin and colleagues identified two viruses present in nematodes infecting the livers of wild mice, corresponding with severe liver pathology [32]. In the *co-housed model*, we uncovered a narnavirus, which likely infects cryptosporidium, which can infect and transmit between mice [9]. Impacts of these viruses on their host and on the pathogenicity of the parasite are unknown.

The study of the wild-mouse immune systems is quite challenging, due to their outbred nature, diverse environmental histories, and range of ages at trapping. The immune systems of wild mice vary greatly in steady-state levels of innate and adaptive immune cell numbers, serum antibody levels and faecal concentrations of IgA, but much of that diversity is not due to genetics or microparasite infection [33]. Age, body condition (scaled mass index), adipose tissue weight and serum leptin concentrations were important drivers of the immune state [33]. Generally wild-animal immune cells are more responsive to stimulation, but often display greater heterogeneity of responses compared to inbred laboratory mice [34, 35]. Wild mice have more matured immune systems including increased serum antibodies, memory T cells, germinal centre B cells, highly activated myeloid cells [36] and increased numbers of NK cells [37]. Wild mice have higher levels of serum antibodies, including 10–100 times more serum IgE [36, 38]. Wild mice also have a thicker mucus layer lining the gut epithelia than laboratory mice [39] – this increased barrier may impact subsequent enteric infections.

Studying wild mice has several benefits, particularly that the animals are living in their natural habitat, foraging for food, and are exposed natural mouse pathogens. However, these animals are outbred preventing the use of modern genetic tools. There are also many variables that are difficulty to control for. The animals need to be properly identified, either through sequencing or visual inspection, to confirm species. It is difficult to know the age of wild mice. Some studies use mouse length (tip of nose to base of tail) [2, 40, 41], a combination of length and weight [38], or eye lens density to determine age [33, 36, 42]. Additionally, studies are limited by animals that are caught in traps, which can be impacted by geography and climate.

## Co-housing

To introduce natural murine micro-organisms, laboratory SPF animals are co-housed with mice from pet stores. Pet store mice live in large colonies and are exposed to a variety of natural mouse pathogens including bacteria, helminths, fungi and viruses [8, 9, 43–47]. The microbiota and pathogens can be transferred from the pet store mouse to laboratory mice at physiological doses through natural routes of transmission. With most experiments in this model, co-housing occurs for 60 days. To screen for the impacts of co-housing, mice are bled for PBMC immunophenotyping and evidence of prior infections by serology [44–47]. CD4^+^ and CD8^+^ T cells from the blood of co-housed mice upregulate CD44, a marker of T-cell activation [44–48]. The level of T-cell activation in the blood and secondary lymphoid organs of co-housed mice is similar to adult humans [44, 49]. The serology panel tests for 18 mouse pathogens. In our experiments using hundreds of laboratory mice co-housed with mice from three separate pet stores over a 3 year period, we have identified 153 unique pathogen combinations [45]. Importantly, no specific pathogen combination correlated with the increase in CD8^+^ T-cell activation [45]. Additionally, over time the microbiome in co-housed mice more closely matches pet store mice than SPF mice suggesting engraftment and equilibration with a more natural microflora [46]. These data suggest the impacts on basal immune responses are not due to any individual microbe or microbe combinations but instead reflects the immune system responding in general to a more diverse microbiome. Additionally, independent groups from different universities have been able to replicate the phenotypes of this model [50, 51]. Together, these features demonstrate that the key factors of the co-housing model are consistent, robust and reproducible across institutions [44–46, 50, 51].

Deeper analyses have demonstrated the immune system is altered in co-housed mice and the resting immune transcriptome of co-housed mice is more similar to adult humans [44]. Additionally, basal cytokine and chemokine levels are elevated in co-housed mice [44]. These changes result in a more robust response to *Listeria monocytogenes* bacterial challenge [44, 46]. Conversely, the response to influenza A virus (IAV) infection was not different between SPF and co-housed mice [45]. However, the vaccine transcriptional response signature of co-housed mice is also more similar to adult humans [45]. When evaluating the response to IAV vaccines SPF mice overexaggerated vaccine responses and co-housed mice were less protected against IAV challenges [45]. It is interesting to note the overall levels of innate and adaptive immune cells in peripheral blood, secondary lymphoid organs and non-lymphoid tissues are much higher in co-housed mice than SPF mice [44, 46, 51–53]. The numbers of immune cells at sites of infection or vaccination may greatly impact responses, highlighting the importance of studying vaccination in mice with mature immune systems.

One major benefit of the co-housing system is the ability to maintain laboratory mouse strains and the tools of genetically defined mouse research. Pet store mice are outbred and tools such as CD4^+^ and CD8^+^ T-cell tetramers cannot be used. By co-housing C57Bl/6 mice, established immunological tools can be used. The co-housing model also presents several disadvantages both logistical and biological. It is important to note the co-housing model must be performed in a facility separate from SPF housing to prevent introduction of micro-organisms to the SPF facility. Measures must be in place to minimize the risk of pathogen transfer. These should include directional airflow to reduce airborne spread of pathogens outside the co-housed facility, dedicated caging and cage sanitation equipment and education of staff for order of animal room access. The best option at our institution was a biological safety level (BSL) 3 facility. Though this far exceeds the level of containment needed to work with these mice, our SPF facilities are protected from contamination from the co-housed facility. Due to ethical constraints males cannot be used for co-housing. However, to address sex as a biological variable, males can be housed with contaminated bedding from pet store mice, which drives similar pathogen exposure and impacts on the immune system as co-housing [45, 47].

The co-housing system may be viewed as artificial because so many diverse microbes and pathogens are introduced at once. We lose on average 22 % of mice during the co-housing period, necessitating larger starting numbers of co-housed animals [44]. However, free-living rodents and humans have been found to harbour many pathogenic eukaryotic viruses at the same time suggesting that exposure to many acute and chronic pathogens at once is common in nature [2, 7, 9]. Another potential source of artificiality is that in this model, animals receive their first infections as adults, instead of during childhood as in the wild. One option to overcome this caveat is to co-house juvenile animals instead of using adults. A more natural life history would include exposure to microbes and pathogens throughout life, instead of a single exposure event.

Another complication in this system is not knowing what microbes are transmitted. To further evaluate the dynamics of virus transmission in this model we co-housed wild-type C57Bl/6 and interferon receptor-deficient mice with pet store animals and evaluated the virome by deep sequencing. We found transmission of many virus families, detected viruses not normally screened for in vivaria, and uncovered novel eukaryotic viruses [9]. As mentioned above, the T-cell activation phenotype is a general response to a diverse microbiota, and not specific to a given pathogen [45]. One of the strengths of this model is the diversity of micro-organism exposure, which mirrors the human experience, though it can be challenging to embrace the chaos.

## Transfer of wild gut microbiome

In this model germ-free laboratory mice were reconstituted with intestinal contents from wild caught mice [40]. The intestinal contents that were chosen as donor material were negative for banned SPF pathogens including 22 viruses, 30 bacteria and 16 meiofauna. Transfer of the wild intestinal contents drastically changed the microbiota of formerly germ-free mice to closely correlate with wild mice. The microbiota was stable in the offspring of these mice through four generations. While the bacterial microbiome was well characterized in this model, it is unknown which viruses, if any, were transferred. As discussed above there are many viruses commonly found in wild and pet store mice that are not routinely screened for in SPF panels.

Introduction of wild microbiota can impact the response to pathogenic infections. Mice reconstituted with wild microbiota were protected from lethal IAV challenge, unlike SPF laboratory mice [40]. Wild microbiota reconstituted mice had reduced induction of chemokines and cytokines after IAV infection. Hyperactivation of inflammatory cytokines and chemokines similar to a cytokine storm has been noted in humans with lethal IAV infections [54–56]. These data demonstrate that introduction of diverse natural microbiota into germ free mice can dramatically impact the immune system at baseline and the response to pathogenic infections.

This model has several advantages: the wild microbial flora is stable for multiple generations, research can be performed in SPF facilities, and established mouse genetics and associated tools can be used [40]. Additionally, animals are exposed to natural flora as infants, more closely mimicking the natural history of free-living animals. While viruses were not formally assessed this could easily be incorporated. This model also has some drawbacks. While incorporating natural flora this model only maintains microbes that are vertically transmitted and likely excludes many viral pathogens and routes of transmission.

## Foetal transfer to wild pseudopregnant females – ‘wildlings’

Rosshart and colleagues expanded on the transfer of the wild gut microbiome model by implanting pseudopregnant wild mice with conventional C57Bl/6 embryos [41]. The pups born to these wild mice are termed ‘wildlings’ [41]. The gut, skin and vaginal microbiome of laboratory, wild and wildling mice was assessed by 16S rRNA, internal transcribed spacer 1–2 rDNA and next-generation virome sequencing to assess the bacterial microbiome, mycobiome and virome, respectively. The bacterial microbiome of wildlings is most similar to wild mice, with more diversity in both bacterial phylum and family than conventional mice. Increased fungal DNA reads were observed in wild and wildling mice. Both mouse viruses and phage reads were elevated in wild and wildling mice. Most laboratory mice had numerous phage reads, but little to no eukaryotic viral reads. The few laboratory mice that had eukaryotic viral reads exhibited minimal viral diversity. Both wild and wildling mice harboured a variety of viruses from multiple families, including Astroviridae, Picobirnaviridae, Circoviridae and ssDNA viruses [41]. The microbiota (bacteria, fungi and viruses) was stable in the wildlings for at least five generations. A deep analysis of the immune systems of these mice demonstrated a transcriptional profile similarity between wild and wildling mice.

The wildling system was used to predict the efficacy of drugs in humans. Both CD28-superagonist (CD28-SA) treatment and anti-tumour necrosis factor (TNF) or TNF receptor:Fc fusion protein (TNFR:Fc) treatment demonstrated promising results in SPF animals, but catastrophic failures in humans [3, 4, 57–59]. CD28-SA treatment in SPF rats resulted in expanded anti-inflammatory T regulatory cells (T_regs_), but in humans led to activation of inflammatory T cells and cytokine storm [3, 57, 58]. Like humans, treatment of wildling mice with CD28-SA did not induce T_reg_ expansion but did increase cytokine levels. Anti-TNF or TNFR:Fc treatment during septic shock in SPF mice protected mice from death, but in humans and wildling mice increased mortality was observed [4, 41, 59]. These studies demonstrate the need for pre-clinical testing to also occur in mice with diverse microbiota and mature immune systems.

There are several benefits to the wildlings model. Similar to the *transfer of wild gut microbiome, wildlings* can exploit established mouse genetics and associated tools. The wildlings model also results in microbial exposure from the beginning of life. There are several drawbacks to this approaching including requiring a surgery, which could increase both cost and labour and an exclusion of pathogens transmitted through non-vertical routes. Similar to the *co-housing model*, *wildling* mice must be housed separate from standard SPF facilities [41, 47].

## Rewilding

In addition to direct exposure from other mice, wild mice are also exposed to microbes in the air, soil and food. In an attempt to add back natural ecological microbes, SPF C57Bl/6 mice were released into natural, farm-like habitats. Rewilded mice lived in these environments, in the absence of wild rodents, for 2–6 weeks before they were captured and assessed for changes in the microbiome and susceptibility to nematodes [60]. Compared to laboratory mice, the rewilded mice had a more diverse microbiota and were more susceptible to nematode infection [60], highlighting the importance of natural microbiota in infection studies.

Subsequent studies using this model expanded on the impacts to the immune system and microbiota. Rewilded mice have increased activation of blood CD8^+^ T cells, similar to the *co-housing model* [44, 45, 61]. Rewilded mice also have increased levels of CD8^+^ T cells in the small intestine, as noted in the *co-housed model* [51, 52, 61]. Granulocyte numbers are also increased in rewilded mice in both peripheral blood and the gut draining lymph node, also noted in the *co-housed model* [44, 46, 61]. Environmentally acquired immunomodulatory fungi were found in the faeces of rewilded mice, which were responsible for the increase in granulocytes [61]. Similar to the faecal transfer experiments of Barbara Rehermann’s group, transfer of cecal contents from rewilded mice to SPF mice resulted in immunological changes in recipient mice, including increases in granulocytes [40, 61]. The rewilding model was used to understand the environmental and immune cell contributions to inflammatory bowel disease (IBD) [62]. The environment was the major driver of changes in the immune system, while mouse genetic mutations (*Nod2* and *Atg16l1*) contributed to changes in cytokines [62]. These data demonstrate the complex interplay of commensal microbes and genetics in disease pathogenesis and highlight reasons why natural infections should be incorporated into disease models.

Similar to the other models discussed, rewilding can use established mouse genetics and associated tools. This model also provides a more natural diet than irradiated feed commonly used in SPF facilities. One drawback to this model is constructing and maintaining the outdoor enclosure and depending on location climate restrictions. Not all released animals are recaptured necessitating larger starting numbers. Finally, because rewilding in these outdoor enclosures excludes mice and other rodents, this model therefore generally lacks introduction of natural rodent viruses.

## Co-housing with wild mice in pens – feralized

In a hybrid model between *co-housing* and *rewilding*, laboratory mice were housed in indoor pens with wild mice, termed feralized, for 8, 9 or 14 weeks [63, 64]. In addition, these mice were also exposed to natural soil containing faecal contents from pigs, cows and horses and natural sources of food such as oat and carrot sprouts. Like other models, the bacterial microbiota of the feralized mice underwent changes to resemble wild mice, including increased richness and diversity of families [63, 64]. Additionally, the immune systems of the feralized mice were more activated than laboratory mice, similar to the *co-housed* and *rewilding models* [44, 45, 61, 63].

To date, the virome of feralized mice has not been analysed. A serology panel testing the presence of antibodies against common mouse viruses identified transmission of minute virus of mice and mouse parvovirus to feralized mice [63]. Gross examination of the intestines identified eggs and worms in many of the feralized mice [63]. One interesting aspect of this study was the two co-housing arrangements. In one experiment, wild males were co-housed with laboratory females, in the second experiment, both wild and laboratory mice were female [63]. Feralized mice living with wild males harboured more viruses and worms than feralized females living with wild females. The increased transfer of pathogens in a male–female pen may be due to intimate contact between mice during mating. While pregnancy induces potential variability to the immune system, the feralized model is the only model that supports natural transmission of micro-organisms through sex.

Similar to the other models discussed, co-housing with wild mice in farming pens allows for the use established mouse genetics and associated tools, exposure to natural mouse pathogens, and exposure to aspects of natural housing. Not every institution has access to large indoor pens, which may prevent widespread adoption. As mentioned above, it can be difficult to obtain wild mice due to geography and climate, and wild mice must be properly identified before co-housing.

## Sequential infection

A more reductionist model to introduce immune experience is to sequentially infect laboratory mice with known pathogens. Laboratory mice were infected with two herpesviruses, influenza virus and helminths [65]. Similar to the *co-housed model*, sequentially infected mice exhibited matured immune systems and had resting immune signatures mirroring co-housed mice and adult humans [65].

Because of the reductionist and controlled nature, this model is the easiest to widely adapt. This model also can leverage established mouse genetics and associated tools and can be performed in traditional BSL-2 vivaria without the need for high containment or special nonstandard housing. Additionally, because the pathogens used are known it will be easier to determine pathogen-specific impacts on phenotypes. The sequential nature of this model does add considerable time, as 15–22 weeks are required to set up animals for experimental use. Current iterations of this model use non-natural mouse pathogens, which could drive non-physiologic responses. However, this model could be redesigned to only include natural infections.

## Cross-comparing approaches to increase microbial experience

Each of the different models described above have their own strengths and weakness (Table 1). To be able to better compare results across models and laboratories we suggest performing a panel of assays to assess immune activation, microbial exposure and composition. In Table 1, we listed if each model had published serological results for common mouse pathogens. Most mouse models used the Charles River Assessment panels using either multiplexed fluorometric immunoassays or PCR detection [40, 41, 44, 45, 60, 61]. Table 2 lists the frequency of viruses detected in each model. In future studies, we suggest using similar SPF serology panels and adding in the most commonly found viruses from wild and pet store mice including murine astrovirus 1 and 2, murine Kobuvirus, and alpha coronaviruses [2, 8, 9]. Viruses lack a consensus region of their genomes, like 16S for bacteria, hindering rapid and cheap sequencing-based approaches for virus identification. However, targeted arrays like Virocap and Virochip could also be incorporated on RNA from tissues at terminal time points or from faeces at any time [66, 67]. To assess the impact of microbial exposure on immune response we suggest analysing activation status of CD8^+^ T cells (CD44^hi^ and KLRG1^+^), CD4^+^ T cells (CD44^hi^), and the composition of innate immune cells (CD64^+^ monocytes and Ly6G^+^Ly6C^int^ neutrophils), listed under immune phenotyping in Table 1. Including serology and immune phenotyping data for each of the models will permit further assessment of the strengths and limitations of each of these models and will help to identify microbial factors impacting the immune system.

**Table 1.** Key characteristics of each model. Immune phenotyping is defined as assessing the frequency and activation status of innate and/or adaptive immune cells after generation of the mouse model, but before experimental treatments, such as pathogen challenge. Serology is defined as testing for the presence of common mouse pathogens after generation of the mouse model. Virome is defined as investigating the presence of viruses in the system and/or how these viruses impact the model.

Model	Advantages	Disadvantages	Immune phenotyping	Serology	Microbiome	Virome	References
Wild	Mice in natural environment Exposed to natural mouse pathogens at physiological routes and doses	Outbred mice Identification required Challenging to determine age	Yes	Yes	Yes	Yes	[2, 10, 27–33, 35–38, 40, 41, 44, 63, 64]
Co-housing	Natural mouse pathogens Physiological exposure, routes and doses Maintain mouse genetics	Separate mouse facility needed 22 % loss of co-housed mice	Yes	Yes	Yes	Yes	[9, 44–53]
Gut microbiome transfer	Wild-mouse microbiota Maintain mouse genetics Stable for multiple generations SPF facilities	Virome not assessed, was excluded Vertical transfer only, missing other transmission routes	No	Yes	Yes	No	[40]
Fetal transfer	Wild-mouse microbiota Maintain mouse genetics Stable for multiple generations	Invasive Costly Separate mouse facility needed Vertical transfer only, missing other transmission routes	Yes	Yes	Yes	Yes	[41]
Rewilding	Maintain mouse genetics Exposure to natural environment	Outdoor facility needed 95 % recovery of mice	Yes	Yes	Yes	No	[60–62]
Feralized	Wild-mouse microbiota Maintain mouse genetics Exposure to natural environment	Separate facility needed Dependent on capture of wild mice	Yes	Yes	Yes	No	[63, 64]
Sequential infection	Maintain mouse genetics Known infection history SPF facilities	Natural infection and transmission routes are missing	No	No	No	No	[65]

**Table 2.** Viruses detected in each model. Percentages of mice tested positive for virus by Charles River Assessment Plus or PCR screening. Serological testing was not published for mice from the sequential infection model. For wild-mice cohousing, independent testing was performed.

Viruses	Pet store	Pet store cohousing	Gut microbiome transfer	Wilding	Rewilding	Rewilding	Wild-mice cohousing
Adenovirus (MAV) Type 1 (FL) and 2 (K87)	7%	5%	0%	0%	0%	0%	0%
Ectromelia (mousepox)	0%*	0%†	0%	0%	0%	0%	nt
Hantaan (HANT)	nt	nt	0%	0%	nt	nt	nt
Lactate dehydrogenase elevating virus (LDH/LDEV/LDV)	nt	nt	0%	0%	nt	nt	nt
Lymphocytic choriomeningitis virus (LCMV)	3%	2%	0%	71%	0%	0%	0%
Minute virus of mice (MVM/MMV)	75%	16%	0%	0%	0%	0%	50%
Mouse coronovirus (MHV)	57%	59%	0%	83%	0%	0%	0%
Mouse cytomegalovirus (MCMV)	1%	0%	0%	50%	0%	nt	0%
Mouse parvovirus (MPV/NS-1)	37%	4%	0%	46%	0%	0%	25%
Mouse Parvovirus-1	36%	16%	0%	42%	0%	nt	nt
Mouse Parvovirus-2	61%	12%	0%	4%	0%	nt	nt
Mouse pneumonitis virus (K)	nt	nt	0%	0%	0%	nt	nt
Mouse rotavirus (MRV/EDIM/ROTA-A)	17%	0%	0%	13%	0%	0%	nt
Murine norovirus (MNV)	71%	71%	0%	67%	0%	0%	nt
Mouse theilovirus (TMEV/GDVII)	64%	81%	0%	0%	0%	0%	nt
Mouse thymic virus (MTV/MTLV)	nt	nt	0%	21%	nt	nt	0%
Pneumonia virus of mice (PVM)	1%	3%	0%	0%	0%	0%	nt
Polyoma virus (POLY)	0%	0%	0%	50%	0%	nt	nt
Prospect Hill virus (PHV)	nt	nt	0%	0%	nt	nt	nt
Reovirus (REO)	2%	0%	0%	0%	0%	0%	nt
Sendai virus (SEND)	53%	7%	0%	0%	0%	0%	nt
Number of animals tested	129	681	10	24	12	10	8
Reference	[44, 45]	[44, 45]	[40]	[41]	[60]	[61]	[63]

nt, not tested.

*15 mice tested,

†13 mice tested.

## Conclusions

Viruses can exert profound influence over immune system development and response to future infections. Eliminating eukaryotic viruses from SPF animals almost certainly has helped with experimental reproducibility by reducing confounding variables. However, this has come at the cost of an underdeveloped immune system and loss of microbe–microbe dynamics. Several exciting new models described here incorporate natural infection exposure. Together, these models will help to improve the relevance and translatability of mouse studies, particularly in virology research.
